# Silicon, a Possible Link between Environmental Exposure and Autoimmune Diseases: The Case of Rheumatoid Arthritis

**DOI:** 10.1155/2012/604187

**Published:** 2012-10-18

**Authors:** Cesar A. Speck-Hernandez, Gladis Montoya-Ortiz

**Affiliations:** Center for Autoimmune Diseases Research (CREA), School of Medicine and Health Sciences, Universidad del Rosario, Carrera 24 No. 63C-69, Bogotá, Colombia

## Abstract

Silicon is one of the most common chemicals on earth. Several compounds such as silica, asbestos, silicone or, nanoparticles are built from tetrahedral units with silicon as the central atom. Despite these, structural similarities, they have rarely been analyzed as a group. These compounds generate significant biological alterations that include immune hyperactivation, production of the reactive species of oxygen and tissue injury. These pathological processes may trigger autoimmune responses and lead to the development of rheumatoid arthritis. Populations at risk include those that constantly work in industrial process, mining, and agriculture as well as those that undergo silicone implants. Herein a review on the main features of these compounds and how they may induce autoimmune responses is presented.

## 1. Introduction

Rheumatoid arthritis (RA) is a common autoimmune disease (AD), characterized by synovial inflammation, autoantibody production, cartilage and bone destruction, and other systemic complications including cardiovascular, pulmonary, and psychological disability. The etiology is unknown although it involves a complex interplay among genetic and epigenetic factors as well as environmental exposure [1].

The influence of several environmental stressors has been broadly described in processes that may trigger autoimmune responses which lead to RA. Habitual smoking and certain previous infections (i.e., *Porphyromonas gingivalis, Epstein-Barr* virus, cytomegalovirus, *Proteus *sp., and *Escherichia coli)* are the most significant associations that have been found for this disease [1, 2].

In the case of a particular class of chemical compounds, long exposure to them has been related to RA, and, in spite of their similar biophysical and biochemical properties, they have rarely been analyzed as a group. This is the case with the silicon-derived compounds (silica, asbestos, silicone, and nanoparticles; for details see Table 1). All of these compounds are built from tetrahedral units with silicon as the central atom and are basically extended networks based on Si–O–Si bonds [3]. In human tissues, silicon is associated with glycosaminoglycans that covalently attach to core proteins to form proteoglycans, which are part of the connective tissue matrix [4].

The goal of this paper is to discuss the chemical features of these kinds of compounds and to describe the biological and immunological alterations that are generated *in vivo. *These alterations may trigger autoimmune responses and lead to autoimmune diseases such as RA. At the same time, another goal is to indicate the different sources of exposure and the population at risk from this class of compound.

## 2. Asbestos

Asbestos are a series of silicate minerals that produce thin fibers when they are crushed; however, this feature covers a large number of distinct minerals. That is why there is an inappropriate and incomplete definition of “asbestos” which makes their classification difficult [5]. The negative effects of asbestos on health were recognized in the early twentieth century. Miners and mining communities are the most vulnerable populations but are better prepared to limit their exposure to asbestos than homeowners who are unknowingly breathing asbestos. Today, it is difficult to associate documented exposure to current symptoms and demonstrate that the diseases in both residents and miners are caused by asbestos due to the large range of periods of exposure (from 15 to 40 years) [6].

Chemical and physical properties of asbestos are related to its carcinogenicity, fibrogenecity, and toxicity. Asbestos is mainly absorbed through air passageways, and the deposition of inhaled fibers in the lung is determined by their length, width, shape, density, and by the anatomy of the respiratory tract [7]. Approximately 20% of elongate minerals inhaled are retained in the respiratory tract, primarily in the tracheobronchial and alveolar areas [8]. The Libby community (Montana) is the community that best depicts the high prevalence of pulmonary disease resulting from occupational and environmental exposure to asbestos. This town has recently been the focus of national attention about the danger of particle silica and asbestos [8].

As it was mentioned before by Aust et al. [9], there are a number of factors thought to induce pathological responses to asbestos. These include levels and intensity of the dose, frequency of exposure, durability of the dust in the biological system, toxicity of a given dust, and individual susceptibility to these variables [9]. Although those pulmonary manifestations of asbestos exposure are well documented, the nonpulmonary outcomes are less understood. One of these manifestations is malign mesothelioma. The relationship of asbestos with immune diseases (specially the risk of developing systemic autoimmune diseases) has been assessed, but it is less conclusive than the relationship with other diseases, and more tests are necessary [10].

### 2.1. Biological Responses to Asbestos

The ability of asbestos to persist over time in the body (mainly in the respiratory tract) is a feature that makes these kinds of compounds a risk for miners and the population associated with them [7]. Asbestos exposure is associated with pulmonary interstitial fibrosis due to accumulation and deposition of inflammatory cells within the lung with subsequent destruction of the lung airspaces. Thus, chemotactic peptides, proinflammatory cytokines, and growth factors produced by lung fibroblast, lung epithelial, and alveolar macrophages are important mediators in the immunological responses against this exposure [11]. For instance, asbestos stimulates the transcription of interleukin-8 (IL-8), which is the major neutrophil chemoattractant in the lung, and the transcription of transforming growth factor-*β*-1 (TGF*β*-1), an important mediator of hematopoietic differentiation, cellular chemoattraction, and stimulation of fibroblast and myofibroblast [11, 12]. Likewise, Uppal et al. [13] found that activated peripheral blood mononuclear cells (PBMCs) of RA patients showed higher levels of IL-8 [13]. In addition, polymorphism in *TGFB1* has been related to bone-erosive damage in these patients [14]. This may indicate the relationship between the outcomes of exposure to asbestos and RA. [13, 14] The results of Song et al. [15] in which TGF*β*-1 promotes the differentiation of synovial fibroblast to myofibroblast seem to support the above. This is the first step in the process that ends in tissue fibrosis [15]. Pulmonary fibrosis and synovial fibrosis caused by TGF*β*-1 may be a link between pulmonary manifestations and an influence on autoimmunity.

The experimental evidence suggests that exposure to asbestos plays a direct role in the activation of NALP3 inflammasome, the release of interleukin 1 beta (IL-1*β*), and inflammatory perpetuation [16]. The relationship between inflammasome activation and IL-1 production has been well documented, and in this context, it is worth noting that IL-1*β* is present in the synovial tissue of animal models and patients with RA, and its ectopic transfer results in a more aggressive disease [17].

In an *in vitro *model with the T-cell line MT-2, it was possible to determine that a lengthy exposure to asbestos is able to alter the expression of more than 139 genes including chemokine receptor 3 (*CXCR3*) and interferon gamma (*IFNG*), which demonstrates that asbestos influences the responses mediated by the Th1 cell population [18, 19]. Interestingly, asbestos exposure mediates the transcription of multiple inflammatory cytokines through the activation of the protein kinase C (PCK) pathway [20]. Even PCK −/− deficient mice exposed to asbestos present a reduction in the clinical manifestations produced by asbestos [20].

Furthermore, asbestos exposure impairs the cytotoxic activities of natural killer (NK) cells and alters the expression of NK-cell activating receptors. This is preceded by the dysfunctional activities of the extracellular-signal-regulated kinase (ERK) phosphorylation pathway [21, 22]. It should be noted that the impaired function of NK cells and the decrease in their activating receptors have also been observed in patients with RA [23].

Asbestos also is a potent stimulator of reactive oxygen species (ROS) production due to the chemical properties of its fibers (particles rich in iron), which can induce the formation of hydroxyl radicals (-OH), superoxide anion (O_2_^−^), hydrogen peroxide (H_2_O_2_), and subsequent ROS release on the part of inflammatory cells (alveolar macrophages and neutrophils). The most important feature is that asbestos fibers cause mitochondrial dysfunction in alveolar epithelial cells (AECs) through iron-catalyzed ROS and final apoptosis of this cellular group [24].

Studies *in vivo* have shown that crocidolite (a particular kind of asbestos) induces a significant increase in mutation frequency, especially transversion of type G-T. This is very probably due to the formation of premutagenic DNA bases such as 8-hydroxydeoxyguanosine (8-OHdG), where free radicals play a significant role in chemical changes on nitrogenous bases [25]. In brief, different studies have provided evidence about the mutagenicity mediated by ROS, which is, in turn, produced by environmental exposure to asbestos and silica particles [26]. The fact that ROS production plays a vital role in the main immune process that leads to an inflammatory process in RA should also be highlighted [27].

### 2.2. Asbestos and RA

In 2006, Noonan and colleagues [28] published a nested case-control study in which 7,307 residents of Libby (Montana) participated. The results showed that this population presented a 65% increase in the risk of developing RA and a 54% increase in the risk of other systemic ADs. Moreover, the OR calculated for the association between asbestos exposure and RA was 3.23. Noteworthy, this population had been exposed to asbestos for over 70 years through mining [28]. In addition, Pfau et al. [29] found that the serum of individuals evaluated in this particular population showed a higher frequency of antinuclear autoantibodies (ANAs), extractable nuclear antigen autoantibodies (ENAs), and a higher serum IgA level compared to other populations with similar geographic and demographic characteristics such as Missoula in the state of Montana [29]. Olsson, in turn, showed that miners who were exposed to asbestos present a higher risk of developing RA [30].

However, despite the fact that Salazar et al. [31] found alterations in the titer of ANAs when Lewis rats were exposed to asbestos, they failed to show correlations with other indicators of RA induction in these mice such as onset, joint inflammation, or RA serum biomarkers (rheumatoid factor (RF) or anti-CCP autoantibodies) [31]. In another case, Pfau et al. [32], with C57BL/6 mice, was able to demonstrate that exposure to asbestos not only increased the levels of ANAs (mainly anti-dsDNA) but also caused glomerulonephritis to develop with a marked complex deposition in the kidneys [32]. Finally, antifibroblast autoantibodies (AFA) were detected in this strain of mice. AFA autoantibodies alter the fibroblast phenotype and stimulate it to differentiate toward myofibroblast and production of type I collagen [33].

The experimental evidence shows that, in spite of the strong and toxic effects on the immune system, the relationship between asbestos and autoimmunity remains unclear. Therefore, and because the epidemiology data suggest a possible association, further research on this issue is warranted.

## 3. Silica

Silica or silicon oxide is one silicon atom combined with two atoms of oxygen (SiO_2_) naturally occurring as quartz or sand. There are multiple crystalline forms and one amorphous form of silica. The continuous inhalation of the crystalline forms of silica has been associated with the development of silicosis, a pulmonary disease characterized by lung pneumoconiosis, diffuse fibrosis, alveolar proteinosis, and loss of pulmonary function [34]. The risk of exposure to these compounds is very high. The majority of activities similar to mining, agriculture, and construction release silica dust, which becomes airborne and puts workers in a position in which they are dangerously exposed [35]. An interesting retroprospective study undergone in a cohort of Chinese workers heavily exposed to silica who were followed for 43 years reported that the main causes of death for 74,040 individuals were related to respiratory diseases, lung cancer, and cardiovascular diseases [36]. However, silica has also been associated with the risk of developing autoimmune diseases, and this is supported by epidemiologic and experimental data.

### 3.1. Biological Responses to Silica

The bioassimilation of silica particles occurs when the particles are coated by phospholipids and surfactant proteins perhaps as a protective mechanism. The cell/particle contact between alveolar macrophages (AM) and silica is the first step in recognition and internalization of silica in the body. After this occurs, it is followed by a marked recruitment of neutrophils and other inflammatory cells through the production of chemokines such as monocyte chemoattractant protein-1 (MCP-1) [34]. The exposure to silica that enters through respiratory passageways causes serious and progressive pulmonary toxicity even after the exposure ceases. The biological effects of silica include direct ones on several pathways such as inflammatory responses, cell-to-cell signaling and interaction, cellular movement that finally leads to cancer and inflammatory and respiratory diseases [37]. Moreover, crystalline silica has been observed to induce more intense responses from gene expression and the cytokine and chemokine secretion than amorphous silica [38].

The toxicity and tissue damage generated by silica in the body involve the production of ROS and nitric oxide (NO). This effect is independent of the length of time the exposure lasts [39], and it is followed by the activation of caspase 3 and caspase 9 with subsequent apoptosis of AM [40]. The effects of ROS produced by silica extend to the ability to produce lipid peroxidation, disrupt lipid rafts, activate protein tyrosine kinase, and to the subsequent translocation of transcription factors such as the nuclear factor kappa B (NF-*κ*B) or the nuclear factor of activated T cells (NAFT) to the nucleus. This leads to the production of several proinflammatory cytokines such as IL-1*β*, IL-8, tumor necrosis factor alpha (TNF-*α*), and interleukin-6 (IL-6) [41, 42].

Moreover, the clearance of silica particles by macrophages leads to NALP3 inflammasome activation, cytokine production, and immune cell recruitment to the affected tissue [16, 43].

### 3.2. Silica and RA

In 1953, Caplan [44] described the occurrence of multiple peripheral lung nodules in coal workers that had RA. This disease was termed Caplan's syndrome. Also called rheumatoid pneumoconiosis (RP), Caplan's syndrome is defined as the combination of multiple well-knit pulmonary nodules which are predominantly on the periphery of the lungs and which are produced by exposure of patients with RA to inorganic silica [44, 45]. The evidence showed that these conditions were highly prevalent in miners with RA. Nevertheless, miners with radiographic signs of pneumoconiosis but without the history and symptoms of RA presented positive levels of RF [46]. As a result, they may possibly develop RA later.

The association between silica and autoimmunity has been assessed (Table 2). Several studies demonstrate that silica exacerbates the development of ADs in genetically susceptible mice models. For instance, Lupus-prone mice exposed to silica showed an increase in ANAs, pulmonary fibrosis, glomerulonephritis accompanied by proteinuria, and circulation and deposition of immune complexes in the kidney [67]. The apoptosis of AM may be a trigger mechanism for autoantibody production and immune complex deposition in this class of mice [68, 69].

Furthermore, mice which have been exposed have higher levels of CD4+ T helper cells, B1 and B cells, and a decrease in regulatory T (Treg) cells followed by an alteration in immunoglobulin levels and an increase in the production of TNF-*α* [70]. Brown Norway rats that were injected with sodium silicate showed higher levels of ANA and ENAs [71]. Further, in a group of workers who had been exposed to silica, the authors observed that two molecules involved in self-tolerance  the cytotoxict-lymphocyteantigen4 (CTLA-4) and programmed death-1 (PD-1) were significantly reduced [72]. The expression of CTLA-4 in Treg cells of patients with RA is significantly reduced and correlates with the abnormal function of Treg cells in this disease [73].

PBMCs and serum from patients with silicosis without clinical symptoms of ADs present higher levels of soluble Fas (an alternative splice of CD95) than membrane Fas [74, 75]. Likewise, decoy receptor 3 (DcR3), which also inhibits interaction between membrane Fas and Fas ligand (FasL) thus affecting apoptosis activation, is overexpressed in these patients [76]. It has been observed that RA patients with active arthritis present higher levels of soluble Fas, and this correlates with markers of the disease activity [77]. The increase in synovial inflammatory cell infiltration in RA has also been associated with the elevated expression of DCR3 [78]. These results suggest that autoreactive cells may escape from apoptosis control for a long time thus leading to autoantibody production and triggering autoimmune responses.

Furthermore, patients with silicosis and other ADs show increased levels of autoantibodies against the death domain of Fas. It is possible that anti-Fas autoantibodies stimulate Fas-mediated apoptosis thus showing another face of Fas in diseases produced by silica exposure [79].

**Figure 1:**
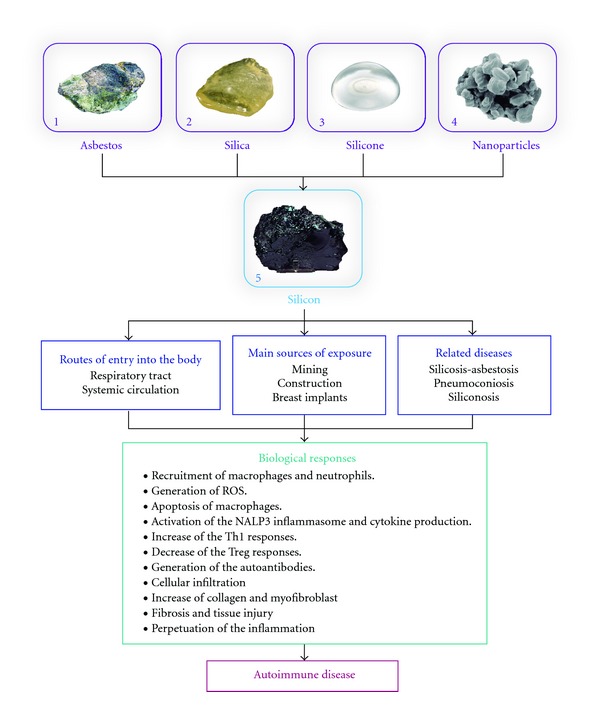
Shared mechanism and biological consequences of exposure to silicon-derived compounds. Figures were downloaded from: (1) natural resources; (2) North east online; (3) Arizona Center for Aesthetic Plastic Surgery; (4) AZoM.com Pty Ltd; (5). Amethyst Galleries, Inc.

Both silica and asbestos can act like superantigens and stimulate polyclonal activation of T cells, which is a mechanism involved in pathogenesis of RA, SLE, and SSc. In the same context, patients with silicosis present significant levels of anti-topoisomerase I, anti-caspase-8, and anti-desmoglein autoantibodies [80–82]. Patients with silicosis also present a reduction in number and function of Treg cells which may be due to activated T cells substituting for them in response to silica exposure [83]. Added to that, patients with RA also present a marked decrease in number and function of this T cell subset [84]. This reduction is mediated partly by Fas which leads the Treg cells to an accelerated apoptosis [83]. Two related compounds (silica and asbestos) generate important molecular and immunological alterations that can function as enhancers of autoimmune responses in RA (Figure 1). Undoubtedly these silicates represent an environmental risk factor for the susceptible population.

## 4. Silica Nanoparticles

Silica nanoparticles (NP) are nanosized structures of silicon dioxide (SiO_2_) and are widely utilized in artificial bones, artificial teeth, interventional catheters, and drug delivery systems. Furthermore, they are used in industries (i.e., paint, catalyst, and textile design) [85]. The cytotoxicity assays showed that the size and porosity of some nanomaterials are an important variable in stimulating inflammatory responses and promote apoptosis [86]. For instance, it has been demonstrated that several classes of NP induce cytotoxic effects such as cell membrane damage, reduction of metabolic activity, generation and release of ROS, apoptosis, and cytokine production in murine macrophages [87, 88].

Strikingly, NPs are able to promote citrullination of proteins such as cytokeratins and plectins through the activation of peptidylarginine deiminase (PAD) [89]. The citrullination of proteins has been related to modifications of antigenicity and production of autoantibodies against these citrullinated proteins [90]. Anti-CCP autoantibodies present high sensitivity and specificity in the diagnosis of RA [91].

Just like silica and asbestos, NP induces the activation of the NRLP3 inflammasome with the release of IL-1*β* and perpetuation of the inflammatory responses as observed in RA [17, 92]. These results suggest a potential mechanism of immune system activation that could possibly lead to RA.

## 5. Silicone

Silicones are a family of silicon oxide polymers that vary in composition based on the length of the polymer and the organic group side chain. When the polymer is short, silicone is a low-viscosity fluid and when the polymer is long, the silicone is a viscous semisolid. The main use of silicone is in esthetic surgery for breast implants, which after decades of research is considered the ideal material for augmentation mammoplasty. For over 20 years, there have been multiple published reports associating silicone breast implants with autoimmune diseases (Table 2) such as RA, scleroderma, morphea, SLE and CREST syndrome [93].

The experimental approaches show that MRL mice −/− implanted with silicone showed increased levels of anti-dsDNA and a modest elevation of RF. Some cytokines such as IL-1 and IL-2 were also elevated [94]. In a murine model of Type II collagen-induced arthritis, the implantation of silicone did not exert any effect on the incidence or severity of the disease. Autoantibodies against silicone-bound proteins were present in the serum of these mice although their pathological significance is unknown. Nevertheless, the long-term implantation of diverse forms of silicone significantly increases the incidence of this animal model of arthritis [95]. Similarly, the genetic background is important in this susceptibility given that the injection of silicone in two different strains of mice—the New Zealand Black (NZB) and BALB/cAnPt (BALB/c)—results in the exacerbation of ADs in one while in the other it does not [96].

It should be noted epidemiological studies have not reported an association between autoimmune diseases, such as RA, and silicone implants. This is also true even with respect to serological markers (autoantibodies) of the disease [66, 97, 98].

## 6. Autoimmune/Inflammatory SyndromeInduced by Adjuvant

A recently denominated autoimmune/inflammatory syndrome induced by adjuvant (ASIA) was defined (for complete review see references [99, 100]). As it was described previously by Shoenfeld and Agmon-Levin [101] this syndrome includes four particular medical conditions, defined by hyperactive immune responses. The major diagnostic criteria are the clinical manifestations such as arthralgia and/or arthritis, neurological manifestations, unrefreshing sleep or sleep disturbances, chronic fatigue, cognitive impairment and memory loss, myalgia, muscle weakness, myositis pyrexia, and dry mouth after a systemic exposure to external stimuli, for example, infections, vaccines, silicone, and adjuvants. There are minor criteria in which specific HLA (HLA DQB1 and HLADRB1) are highlighted and AIDs such as multiple sclerosis (MS) and systemic sclerosis (SSc) are involved [101].

As an adjuvant, silicone is capable of inducing autoimmune-like conditions (e.g., the Gulf war syndrome (GWS), siliconosis, postvaccination phenomena, and the macrophagic myofasciitis syndrome (MMF)). This could be the case for symptoms such as arthralgia and myalgia that are more common in individuals exposed to silicone implants. Siliconosis is one of the most characteristic diseases because of its potential as an adjuvant in the immunization process [99].

Over the last year, a few case reports have related the association of breast implants with autoimmune or autoinflammatory diseases [64, 100, 102]. There are reports that patients with siliconosis began experiencing connective tissue disease (CTD) or immunological syndromes (similar to Sjögren's syndrome (SS), MS, SSc, RA, and others). There seems to be a relationship between siliconosis and CTD. Although siliconosis does not fulfill any diagnostic criteria for a defined CTD, it must be noted that silicosis or asbestosis like siliconosis shared strong immunological and adjuvant responses that could lead to ADs. However there are discrepancies on this issue as found in the meta-analysis study [66].

## 7. Conclusions and Remarks

Environmental factors belong to the large group of significant mediators in the mosaic of autoimmunity. The long exposures to these factors become a risk for specific populations. In this context, silica, asbestos, silicone, or nanoparticles not only generate various immunological alterations but are also extensively in contact with people (Table 1). They may be mediators together with the genetic background in the mechanism that leads to autoimmune diseases such as the case of RA. Furthermore, these compounds are derived from the same chemical group. All of them contain silicon, which is one of the most common elements on earth, and despite their similarities, it is very rare for them to be seen as group. The epidemiological evidence and experimental approach have revealed the role of these compounds in autoimmunity, especially in RA, and their potential in the activation of the cellular recruitment, Th1-Treg misbalance, inflammasome activation, cytokine production, or ROS release. All these responses have been related to autoimmune diseases for years.

## Figures and Tables

**Table 1:** Characteristics and effects of silicon-derived compounds.

Biological Effect	Silica	Asbestos	Silicone	Nanoparticles
Sources	Construction, Minning	Construction, Minning	Breast implants	Nanotecnology, Drug delivery

Inflammation	NALP3 Inflammation	NALP3 Inflammation	No tested	NALP3 Inflammation
	Release of proinflammatory cytokines (IL-1*β*, IL-8, TNF*α*, IL-6)	Release of proinflammatory cytokines (IL-1*β*, IL-8, IFN*γ*)	Release of proinflammatory cytokines (IL-1, IL-2)	Release of proinflammatory cytokines (IL-1*β*, TNF*α*, IL-6, IFN*γ*)

Serology	Production of Autoantibodies (ANAs, ENAs, RF, Anti-Fas, Anti-CCP)	Production of Autoantibodies (ANAs, AFAs)	Production of Autoantibodies (ANAs)	Production of Autoantibodies (anti-CCP)

	Infiltration	Infiltration		Infiltration
Cellular responses	ROS production	ROS production	No tested	ROS production
	Apoptosis	Apoptosis		Apoptosis

	Tissue Fibrosis	Tissue Fibrosis	Tissue Fibrosis	Tissue Fibrosis
Outcome		Perpetuation of the responses along the time		
		Rheumatoid Arthritis		

**Table 2:** Epidemiological evidence about the relationship between silicon-derived compounds and autoimmune diseases.

Compound	results	Reference
	A review of the medical records of individuals reported to the Michigan Silicosis Surveillance system from 1985 to 2006 showed that individuals with silicosis had a two- to eightfold risk of developing RA and SLE with a greater than 24-fold risk for scleroderma and ANCA vasculitis.	[47]
	In a retrospective report of patients attending the Ben Aknoun Hospital (Argelia), 9 cases of autoimmune diseases with occupational exposure to silica were found (7 Si, 4 SSc, 3 RA, 1 SLE, 1 SS).	[48]
	A case control study (577 cases of RA and 659 matched controls) showed that silicaexposure combined with smoking among men is associated with an increased risk of developing anti-CCP positive RA (OR: 7.36).	[49]
	Two out of 78 workers exposed to silica who were evaluated presented positive levels of RF.	[50]
	Association study including 276 male with RA and 276 controls. Of the 276 males cases in this study, 41 were exposed to silica. This exposure increased the 2.2 the risk of RA regardless of age, residential areas and smoking.	[51]
	Case report of a 63-year-old man exposed to silica for 30 years and diagnosed with leukocytoclastic vasculitis.	[52]
	Case report of a 72-year old, a retired dental technician exposed to silica, with a rare case of SS.	[53]
Silica	Case report of a 39-year-old painter who developed severe seropositive RA. Additional investigation revealed silicosis manifested exclusively in the mediastinal lymph node with no pulmonary abnormality.	[54]
	An analysis using death certificates from 27 states in the USA showed an association between potential occupational crystalline silica exposure and mortality due to RA.	[55]
	Case report of a 28-year-old female dental technician showed a history of exposure to ceramic silica and symptoms characteristic of RA with lung interstitial disorder. The patient presented elevated RF and HLA risk haplotypes (HLA-A2-A31, HLA-B51-B18, and HLA-DR3-DR11).	[56]
	A report of two cases of coexistence of pulmonary silicosis and SLE in two men exposed to silica for 20 years.	[57]
	In a morbidity and mortality analysis in a cohort of 4,626 silica-exposed workers in the industrial sand industry, RA was seen to be one of the main causes of death in this cohort (SMR: 4.36).	[58]
	A case-control study to compare the occupational background of 31 cases of biopsy proven vasculitis showed that silica exposure is more present in cases than controls.	[59]
	The report of a case-control study of sixty-five patients with ANCA-SVV and 65 matched control subjects. The results showed that silica dust exposure is associated with ANCA-SVV (OR: 4.6).	[60]
	In a study of 4,500 people in the town of Husavik, a relationship was found between sarcoidosis and exposure to crystallinesilica (OR: 13.2). In 8 cases in which sarcoidosis was found, 6 had been exposed (Iceland).	[61]

	A case series study where three groups of women were compared, the first one developed myositis after they received silicone implants (MASI), the second group was women with myositis but without silicone implants and thelast group was composedof healthy women with silicone implants. This study found that MASI patients have an increased frequency of HLA-DQA1 ∗ 0102 allele.	[62]
	Out of a total of 813 individuals with silicone breast implants, ANAs were found in 244 (30%).	[63]
Silicone	Case report of a 25 year-old female who at the age of 11 was diagnosed with Still's disease. At the age of 22, she underwent silicone breast implant surgery and presented with a transient lupus-like syndrome. Then, at 25 years of age she had a severe activation of Still's disease in association with a rupture of the implants. This case meets the criteria for ASIA syndrome.	[64]
	The impact of implant integrity on clinical symptoms and antibody status was assessed in 90 consecutive female patients with silicone breast implants. The results indicated that implant integrity has no major impact on rheumatic symptoms.	[65]
	A meta-analysis demonstrated that there was no evidence that breast implants were associated with a significant increase in the adjusted relative risk of connective-tissue diseases.	[66]

RA: rheumatoid arthritis; SLE: systemic Lupus erythematosus; ANCA: anti-neutrophil cytoplasmic antibody; SI: silicosis; SSc: systemic sclerosis; SS: Sjogren syndrome; OR: odds ratio; RF: rheumatoid factor; SMR: standardized mortality ratio; SVV: small-vessel vasculitis; MASI: myositis after silicone implants; ASIA: autoimmune/inflammatory syndrome induced by adjuvants; ANAs: antinuclear antibodies.
